# Publisher Correction: N-terminal syndecan-2 domain selectively enhances 6-O heparan sulfate chains sulfation and promotes VEGFA_165_-dependent neovascularization

**DOI:** 10.1038/s41467-019-10205-0

**Published:** 2019-05-07

**Authors:** Federico Corti, Yingdi Wang, John M. Rhodes, Deepak Atri, Stephanie Archer-Hartmann, Jiasheng Zhang, Zhen W. Zhuang, Dongying Chen, Tianyun Wang, Zhirui Wang, Parastoo Azadi, Michael Simons

**Affiliations:** 1 Yale Cardiovascular Research Center, Section of Cardiovascular Medicine, Department of Internal Medicine, Yale University School of Medicine, 300 George Street, New Haven, CT 06511 USA; 2 Complex Carbohydrate Research Center, The University of Georgia, 315 Riverbend Road, Athens, GA 30602 USA; 3 Department of Cell Biology, Yale University School of Medicine, New Haven, CT 06520 USA

**Keywords:** Glycobiology, Angiogenesis

Correction to: *Nature Communications* 10.1038/s41467-019-09605-z, published online 05 April 2019.

The original version of this Article contained errors in Figs. 1, 3 and 4. In panels b and d of Fig. 1, the labels ‘Sdc4^-/-^’ were inadvertently replaced by ‘Sdc4^+/+^’. In panels c and d of Fig. 3, the labels ‘Sdc4^-/-^’ were replaced by ‘Sdc2^-/-^’. In panel f of Fig. 3, the labels ‘FGF2’ were replaced by ‘VEGFA_165_’. In panel e of Fig. 6, a ‘Sdc2^-/-^‘ label was inadvertently included. This has now been corrected in the PDF and HTML versions of the Article.

