# Supplementary material for: N-terminal syndecan-2 domain selectively enhances 6-O heparan sulfate chains sulfation and promotes VEGFA165-dependent neovascularization
Source: Nat Commun. 2019 Apr 5;10:1562. doi: 10.1038/s41467-019-09605-z (PMC6450910; doi:10.1038/s41467-019-09605-z)
Supplement: Supplementary file 1 — Supplementary Information [file 41467_2019_9605_MOESM1_ESM.pdf]

Supplementary information

N-terminal syndecan-2 domain selectively enhances 6-0 heparan sulfate chains sulfation and promotes $VEGFA_{165}$-dependent neovascularization

Corti F. et al

Contents*:*

Supplementary Figures 1-12

Supplementary Table 1 – List of constructs

Supplementary Table 2 – List of primers

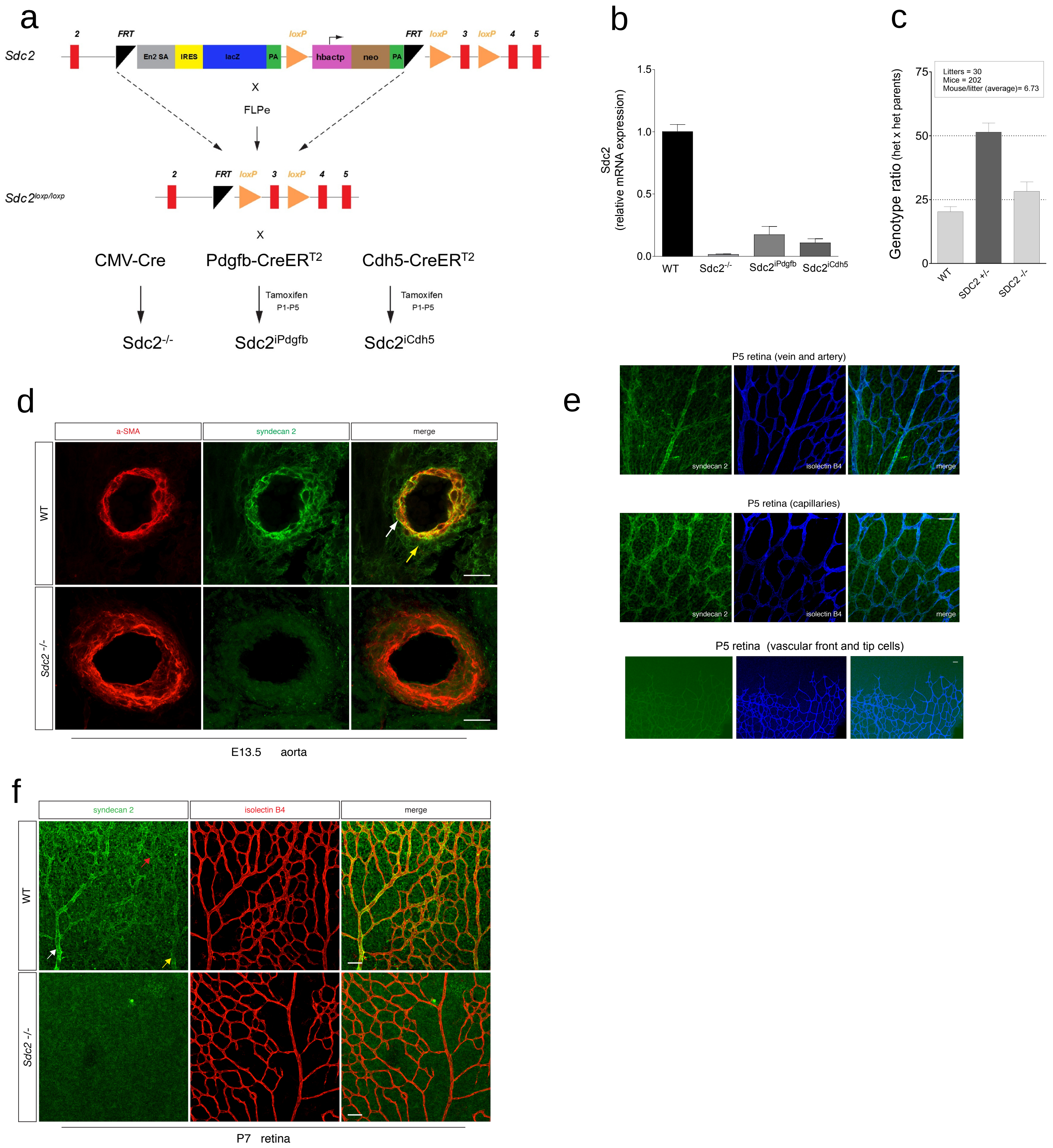

Supplementary Figure 1 | Validation of Sdc2 deletion in vivo (a-f). a Scheme of Sdc2 transgenic allele in ES cells obtained from KOMP repository (Strain ID: Sdc2$^{tm1a(KOMP)Wtsi}$) and crossings to generate various genetic lines used in this study (see Methods for details). **b** qPCR analysis of Sdc2 mRNA level in primary lung ECs after CRE-induced deletion in vivo. **c** genotype ratio of pups obtained from crossing of Sdc2 heterozygous parents. **d** Sdc2 (in green) and α-SMA (in red) staining in section of aorta highlights Sdc2 expression in smooth muscle cells (25 μm scale bars). **e** Sdc2 (in green) and isolectin B4 (in blue) staining in vein and artery (upper panel), capillary bed (middle panel) and tip cells (bottom panel) (50 μm scale bars). Lower panels show tip cell expression. **f** Analysis of Sdc2 expression in P7 retinas from WT and Sdc2$^{-/-}$ mice. Arrows: white - artery, yellow - vein, red – capillaries (50 μm scale bars). Error bars represent standard error of the mean.

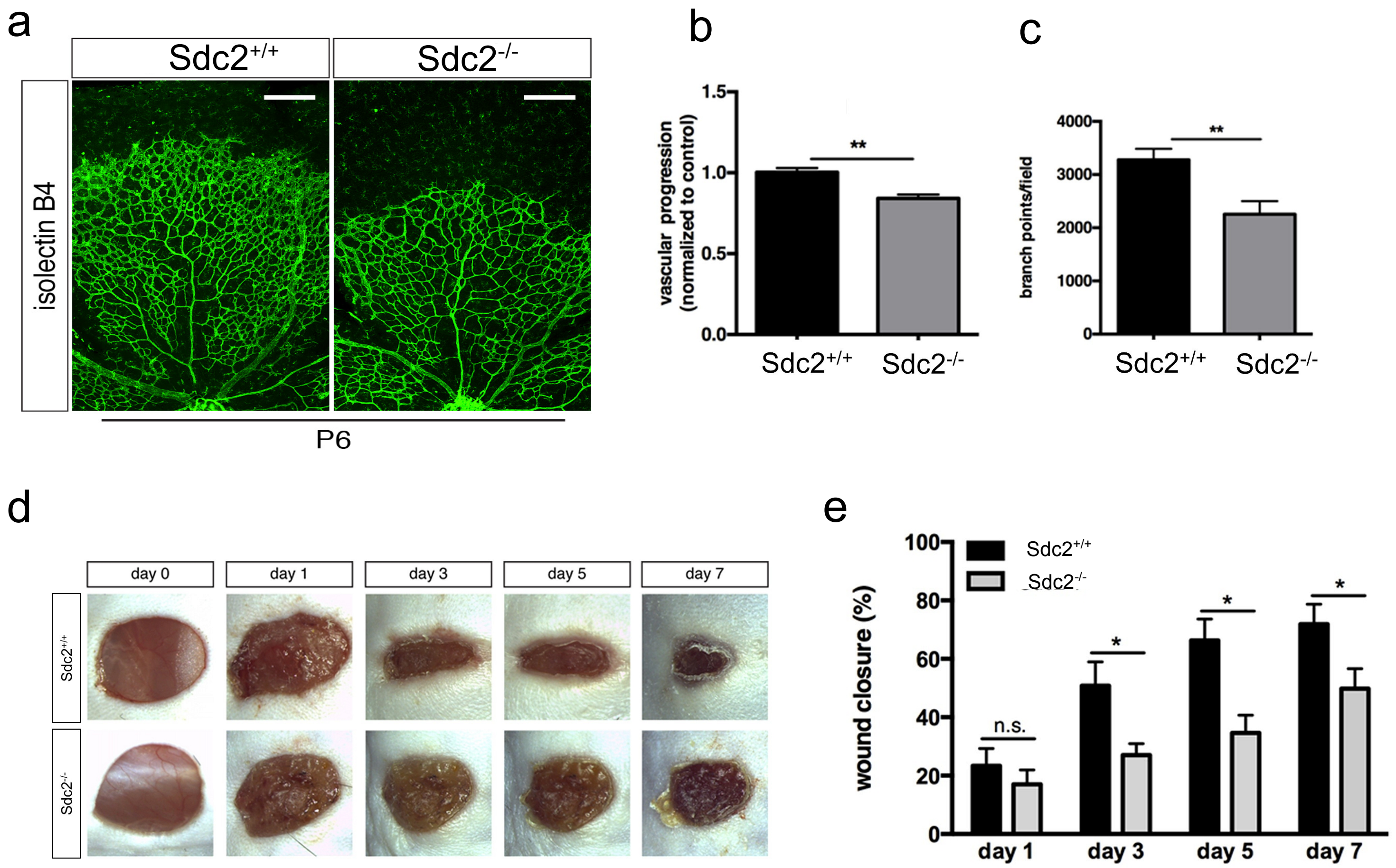

Supplementary Figure 2 | Global Sdc2 deletion leads to live mice with angiogenesis and wound healing defects (a-e). a-c Retinas from P6 pups were stained with isolectin B4 to detect ECs (in green) (250 μm scale bars) and quantify of retinal vascular outgrowth and branching (n=3 retinas from 3 mice, ** P<0.01 by unpaired t-test). **d, e** Delay in skin wound healing is observed in Sdc2$^{-/-}$ mice (n=3, * P<0.05 by one-way Anova with Bonferron's multiple comparison test). Error bars represent standard error of the mean.

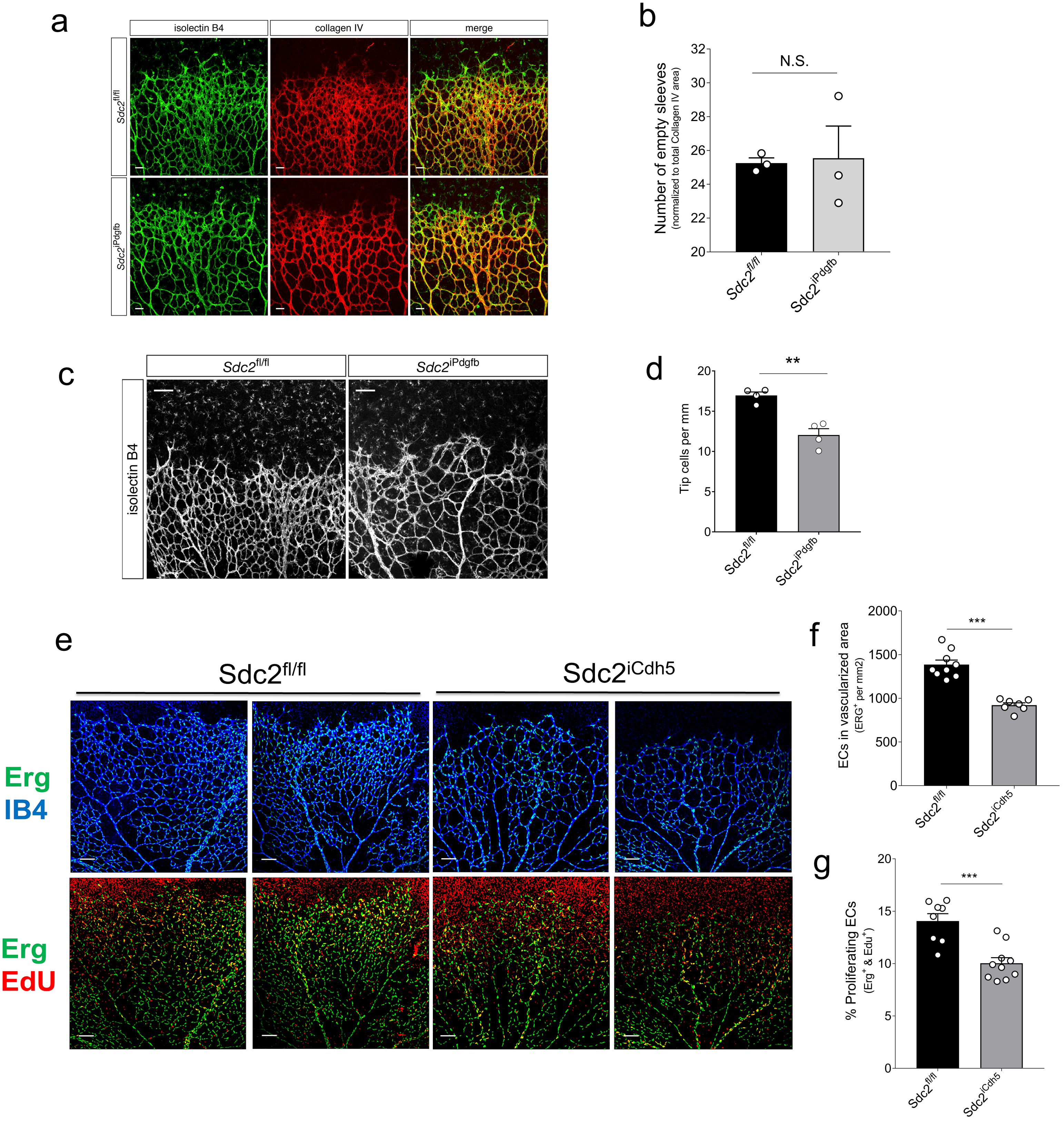

Supplementary Figure 3 | Sdc2 endothelial-specific deletion leads to a delay in vascular development in retina (a-g). a-b Analysis of vascular regression (pruning) in retina. Collagen IV staining (in red) was used for detection of empty sleeves and isolectin B4 for ECs (in green) (**a**, representative pictures with 50 µm scale bars; **b**, quantification) Each dot corresponds to a different mouse (n=3 retinas from 3 mice, ** P<0.01 by unpaired t-test). **c, d** Analysis of tip cells number in P6 pups after Sdc2 endothelial-specific deletion. Isolectin B4 staining was used for endothelial cells visualization (**c**, representative pictures with 100 µm scale bars; **d** quantification). Each dot corresponds to a different mouse (n=4 retinas from 4 mice, ** P<0.01 by unpaired t-test). **e-g** Assessment of ECs density and proliferation with pulse EdU-injection. P5 mice were injected with a single EdU dose and retina was stained after 4 hours (**e**, two representative pictures for each genotype are shown**).** Image J (Cell counter plugin) was used to obtain values of total EC number and double positive Erg/Edu ECs. Each dot corresponds to a different retina (n =8-9 retinas from 4-5 mice for Sdc2$^{fl/fl}$, n=7-10 retinas from 4-5 mice for Sdc2^{iCdh5}, *** P<0.001 by unpaired t-test)**.** Error bars represent standard error of the mean.

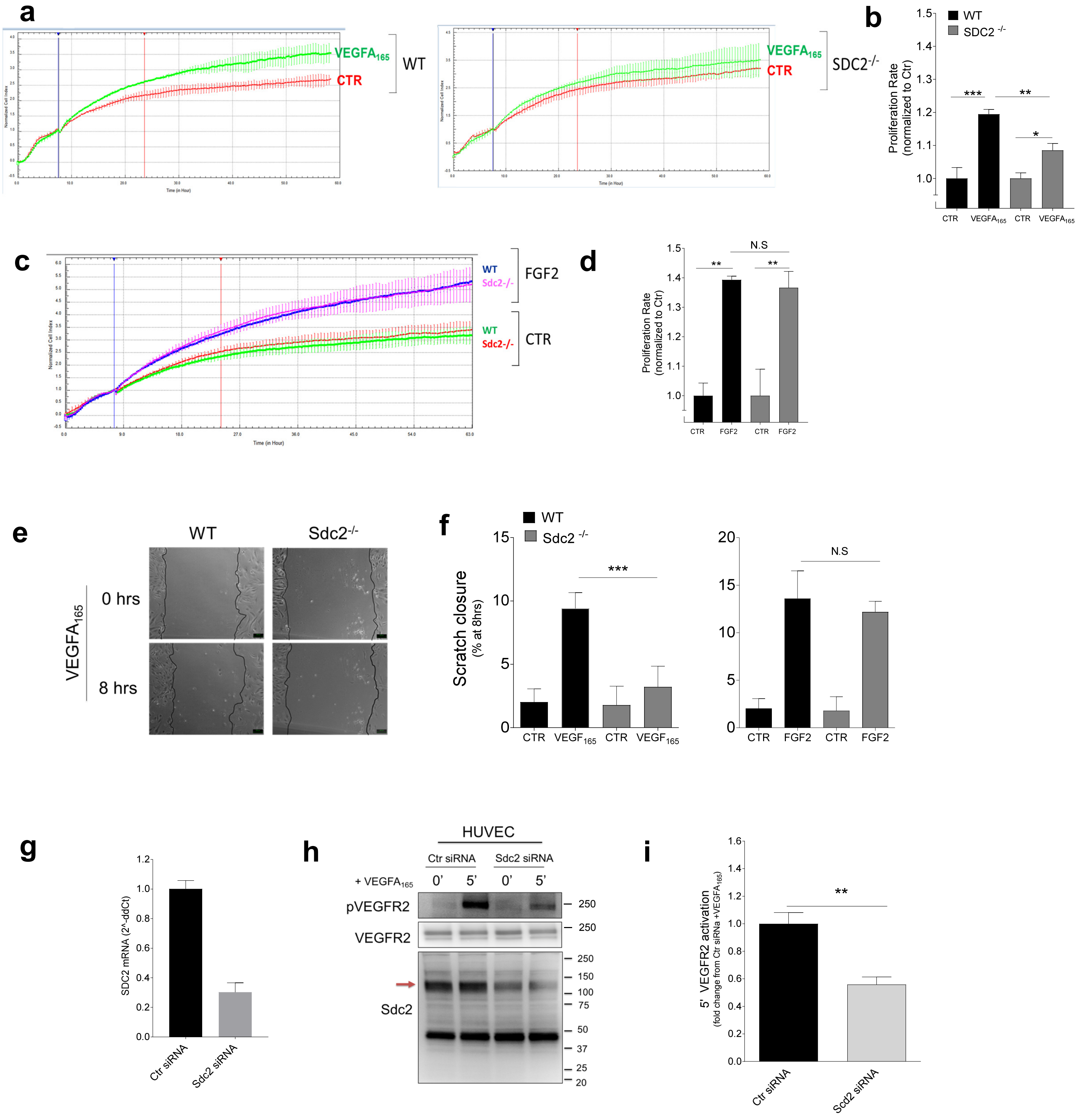

Supplementary Figure 4 | Decreased VEGFA-induced biological effects in Sdc2$^{-/-}$ ECs (a-i), a-d xCELLigence system was used for monitoring real-time proliferation index following growth factor stimulation. Primary ECs were allowed to adhere for 7 hours in 2% FBS then stimulated with vehicle (PBS), VEGFA$_{165}$ (200 ng/ml) or FGF2 (100 ng/ml). Representative pictures of proliferation curves for VEGFA$_{165}$ and FGF2 (up to 60 hours) (**a, c**) and relative quantifications (**b, d**). Blue lines indicate time of VEGFA$_{165}$ stimulation and red line represents 24 hours after stimulation (n=4 independent experiments, * P<0.05, ** P<0.01, * P<0.001 by one-way Anova with Bonferroni multiple comparison post-test). **e, f** Migration was evaluated by scratch assay (**e**, representative picture of VEGFA$_{165}$ -induced closure after 8hrs; **f**, quantification for both VEGFA$_{165}$ and FGF2) (n=4 independent experiments, N.S. not significant, *** P<0.001 by one-way Anova). **g-i** Assessment of VEGFR2 signaling in HUVEC after Sdc2 silencing. qPCR analysis confirming decreased Sdc2 mRNA level (**g**). Activation of VEGFR2 (pVEGFR2) was evaluated following stimulation with VEGFA$_{165}$ for 5 minutes (**h**, representative picture; **i**, quantification) (n=3, ** P<0.01 by unpaired t-test). Red arrow indicates specific band detected by anti-human Sdc2 antibody after treatment of HUVEC with heparinases. Error bars represent standard error of the mean.

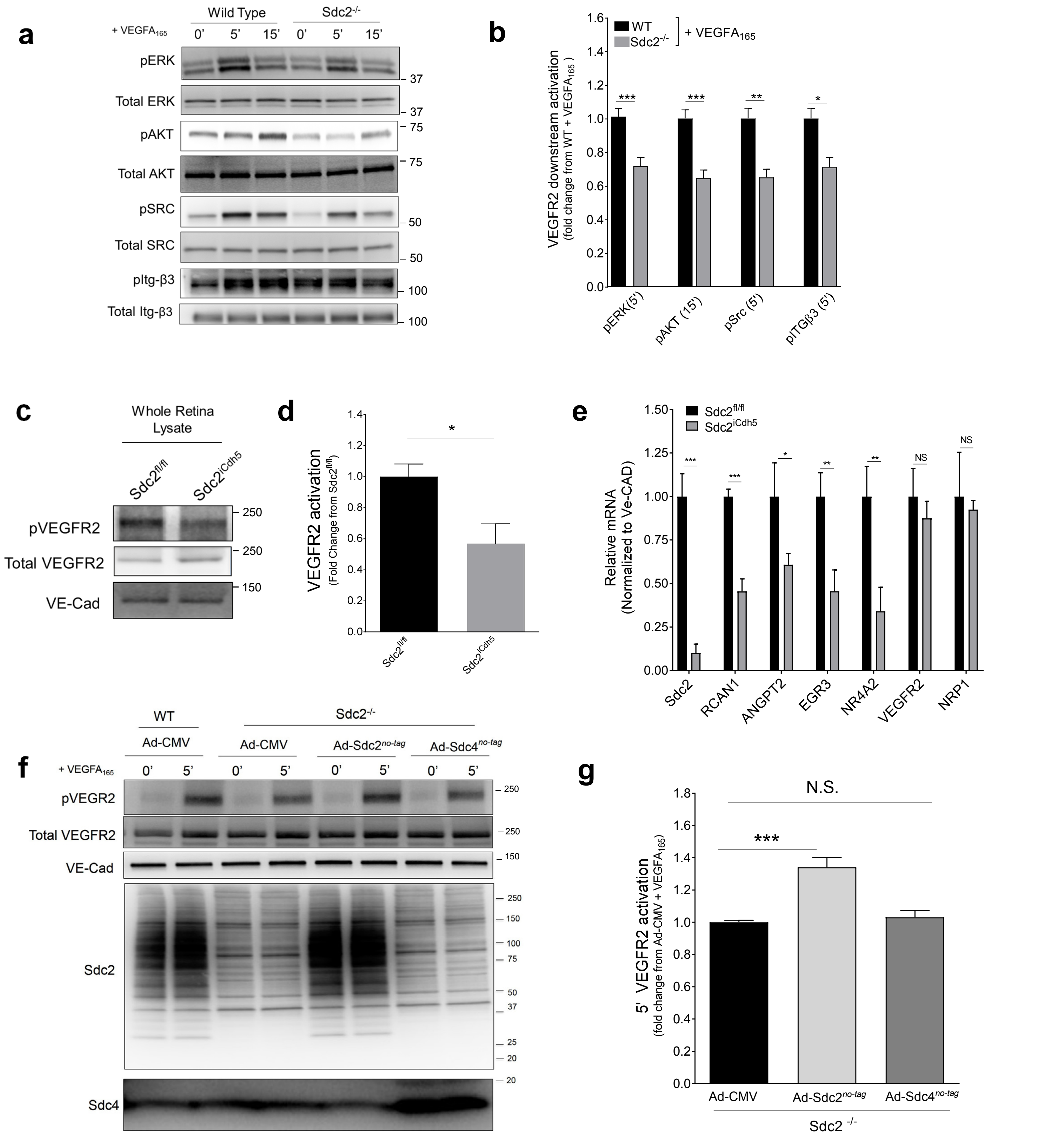

Supplementary Figure 5 | Activation of VEGFR2 downstream signaling in mouse primary ECs and rescue with non-tagged syndecan constructs (a-g). a-c Primary mouse ECs were stimulated with $VEGFA_{165}$ for indicated times and activation of VEGFR2 effectors was evaluated with specific anti-phospho antibodies: pERK, pAKT (T308), pSRC (Y416), pItg (Integrin)-β3 (Y759) (**a**, representative picture; **b**, quantification) (n=4, * P<0.05, ** P<0.01, *** P<0.001, by unpaired t-test). **c, d** Whole retinas from P7 mice were quickly dissected and homogenized in RIPA buffer. Western blot was used for assessment of VEGFR2 phosphorylation (**c**, representative picture; **d**, quantification) (n=4 retinas from 4 mice, * P<0.05 by unpaired t-test). **e** Analysis of VEGFA target genes in freshly-sorted EC from P7 retinas (n=8 retinas from 4 mice for each genotype, N.S. not significant, * P<0.05, ** P<0.01, *** P<0.001 by unpaired t-test). **f, g** Rescue of VEGFR2 in $Sdc2^{-/-}$ ECs with non-tagged Sdc2 and Sdc4. Transduction with an empty adenovirus (Ad-CMV) was used as control (**f**, representative picture; **g**, quantification) (n=3, N.S. not significant, *** P<0.001, by one-way Anova with Bonferroni multiple comparison test). Error bars represent standard error of the mean.

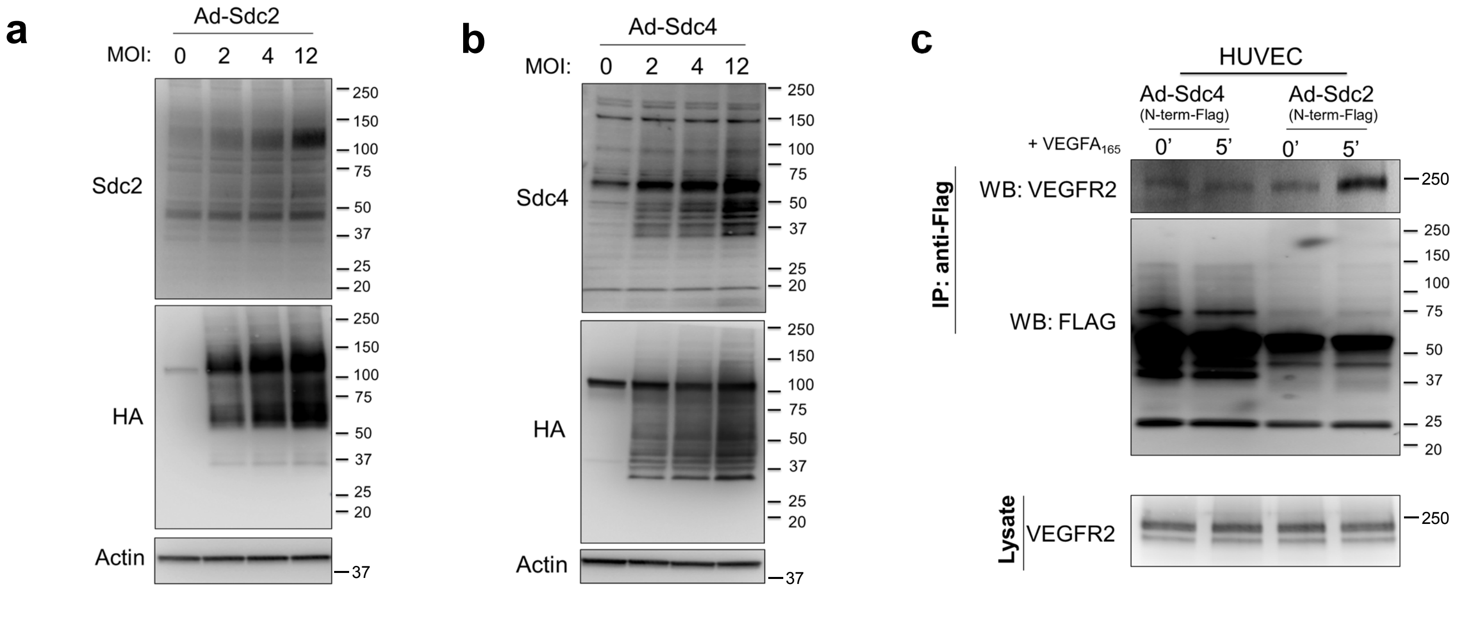

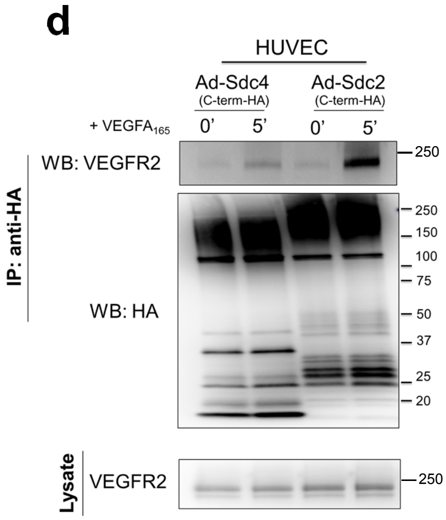

e

	Sdc2	Sdc4
Total Sulfation	52.90 ± 2.749	50.95 ± 2.017
N-Sulfation	44.85 ± 2.443	43.53 ± 2.629
2-O-Sulfation	22.98 ± 1.978	22.2 ± 1.485
6-O-Sulfation	22.90 ± 1.914	15.5 ± 2.545 (*)

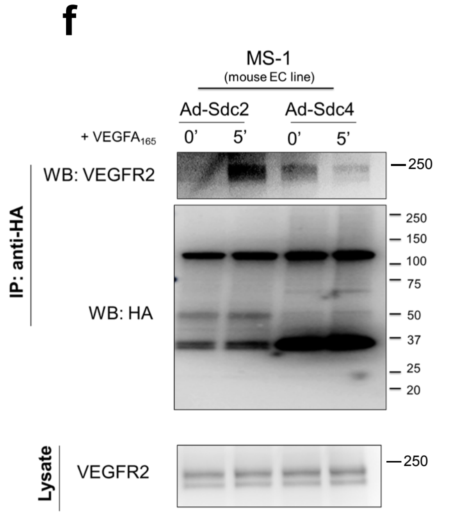

Supplementary Figure 6 | Overexpression levels of syndecan constructs and IP experiments with different epitope-tags (a-f). a-b Western blot showing level of overexpression for Ad-Sdc2 and Ad-Sdc4 at various MOI (multiplicity of infection). MOI=1-2 was used across all experiments of this manuscript. **c-d** Co-IP experiments using syndecan constructs with various tags (**c**, N-terminal Flag; **d**, C-terminal HA). **e-f** Quantification of various sulfation levels in Sdc2 and Sdc4 ED isolated from MS1 cells (**e**) and Co-IP experiments (**f**) (* P<0.05 by unpaired t-test).

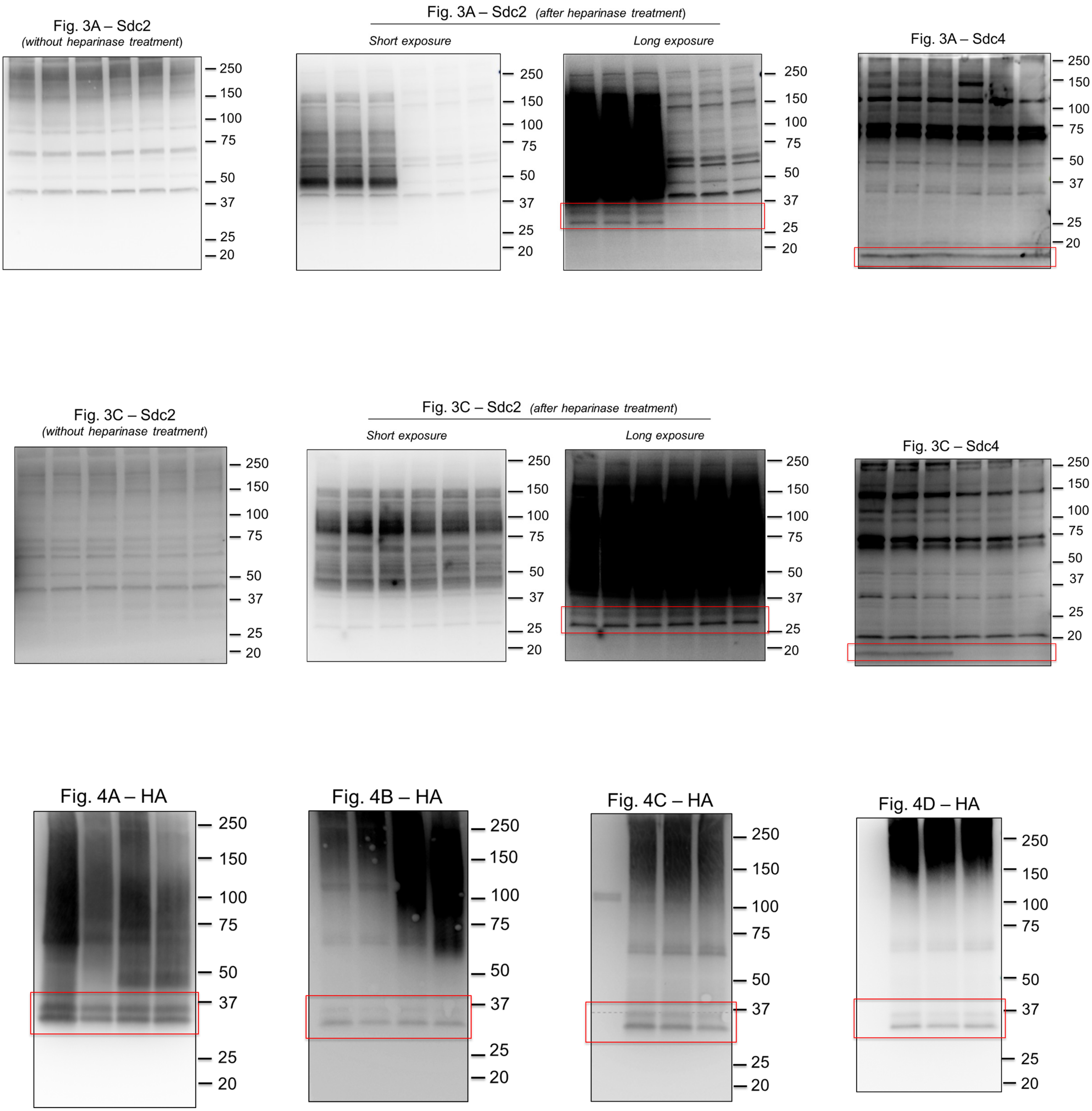

Supplementary Figure 7 | Comparison of Sdc2, Sdc4 and HA expression. Uncropped membranes showing high molecular weight glycosylation smears of Sdc2, Sdc4 and Ha-tagged constructs for figures 3a,c and 4a-d.

Fig.3a

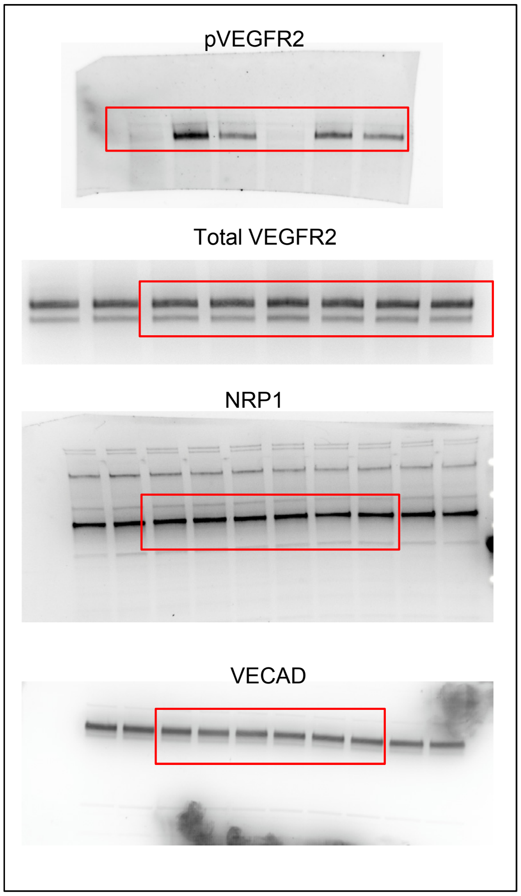

Fig.3c

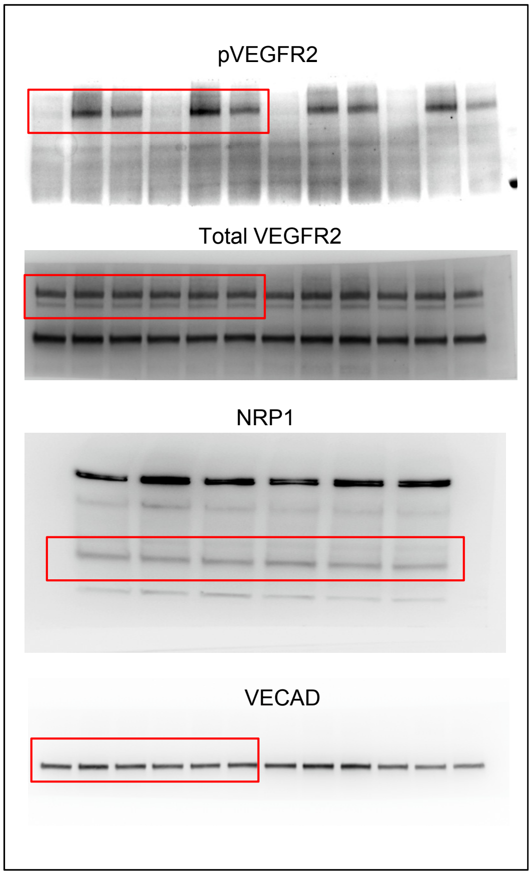

Fig.3e

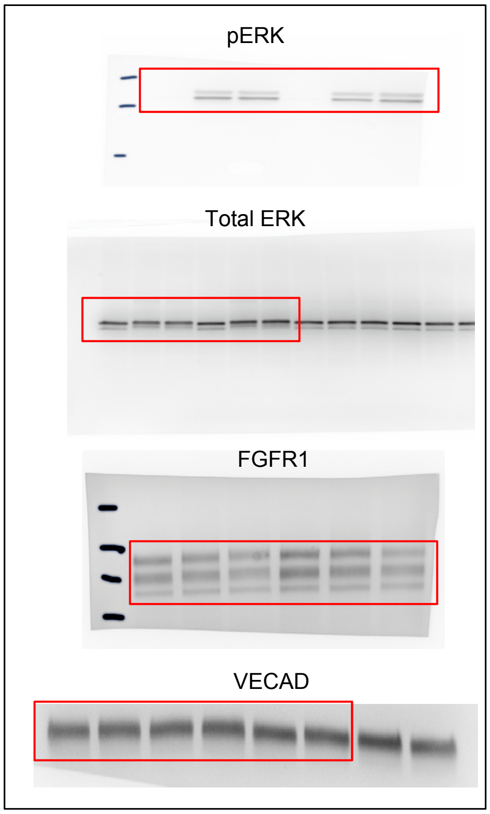

Fig.3g

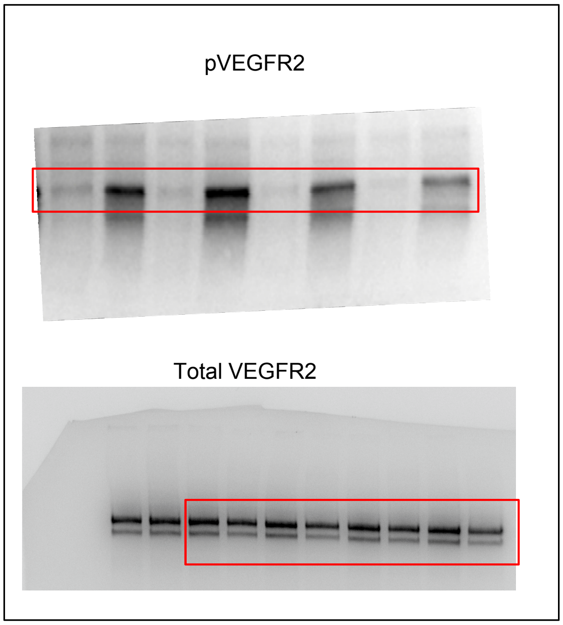

Supplementary Figure 8 | Uncropped western blot images. Membranes for figure 3a,c,e,g

Fig.4a

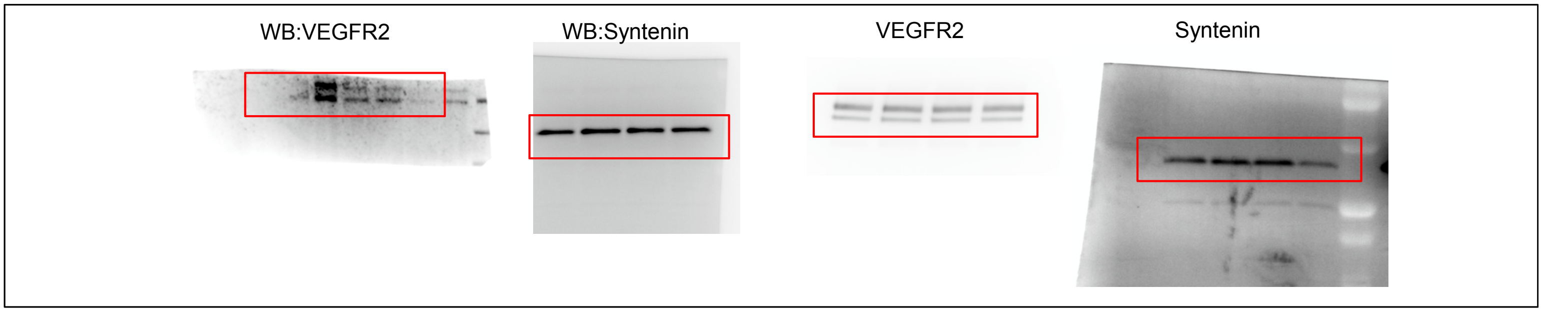

Fig.4b

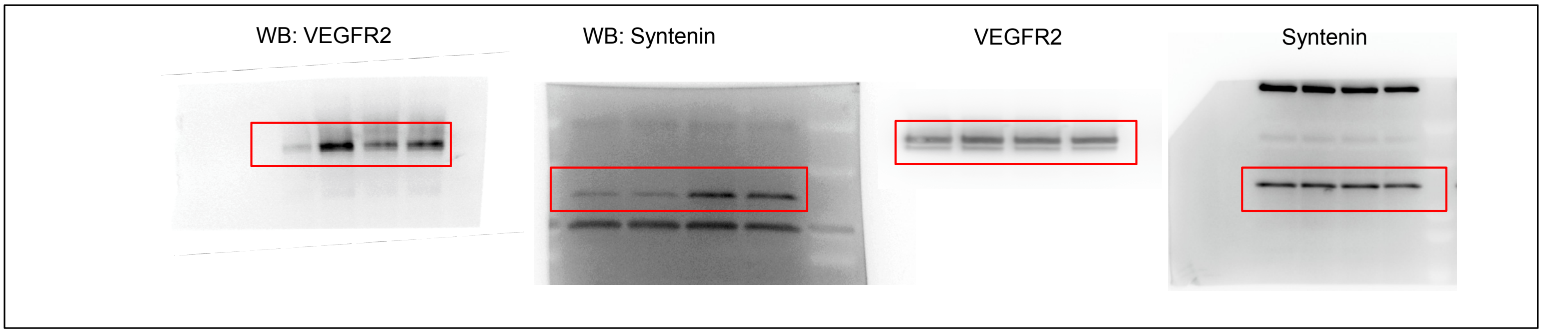

Fig.4c

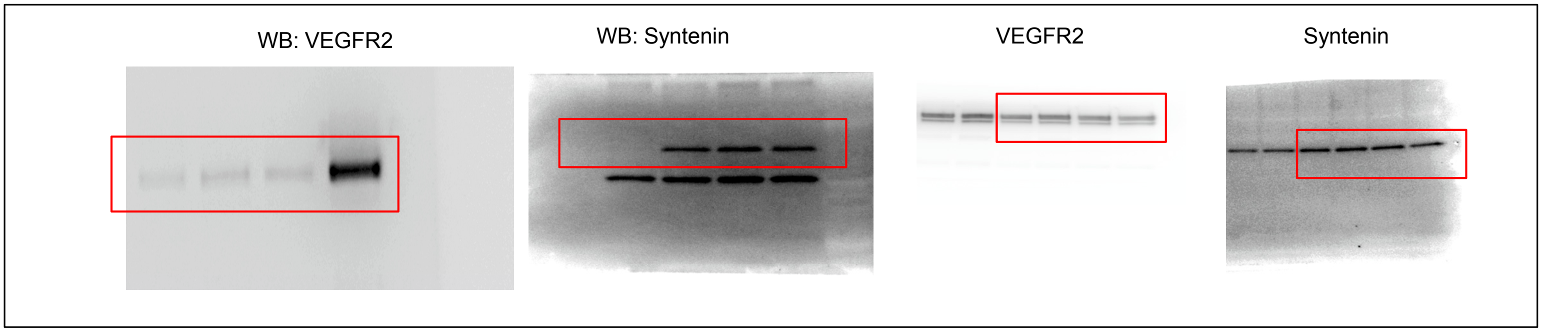

Fig.4d

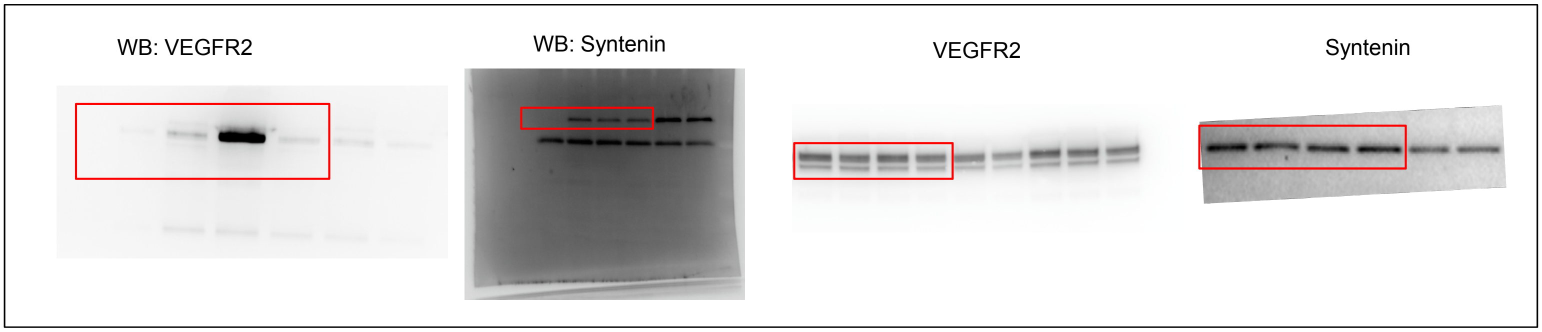

Supplementary Figure 9 | Uncropped western blot images (continued). Membranes for figure 4a,b,c,d

Fig.4e

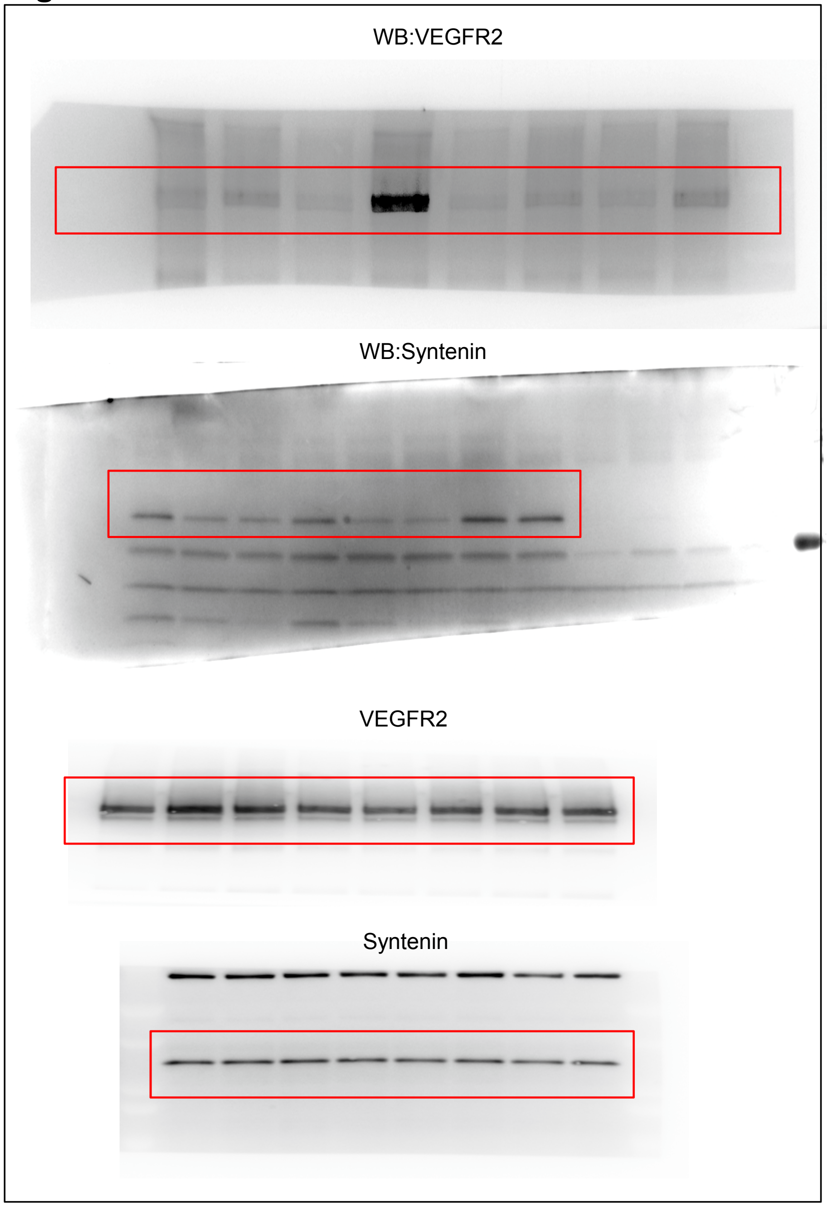

Fig.4f

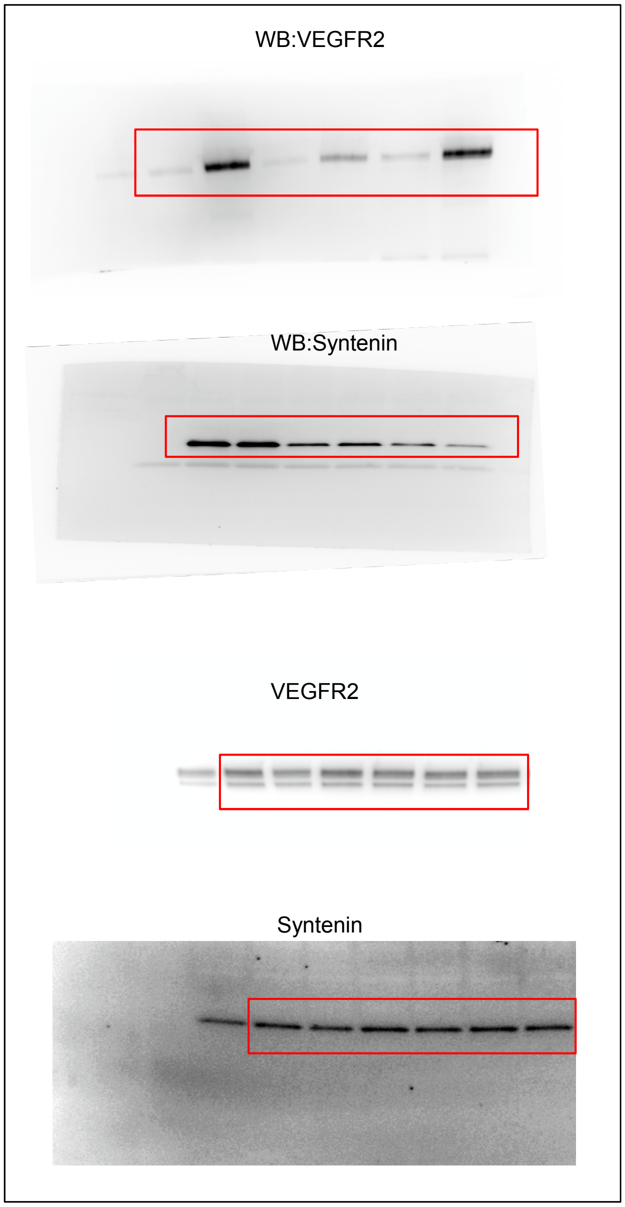

Fig.4g

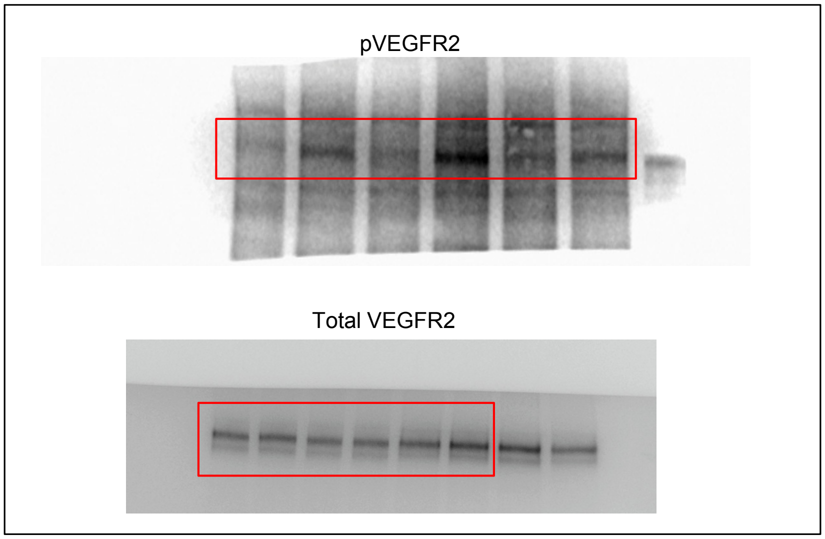

Supplementary Figure 10 | Uncropped western blot images (continued). Membranes for figure 4e,f,g

Fig. 5e

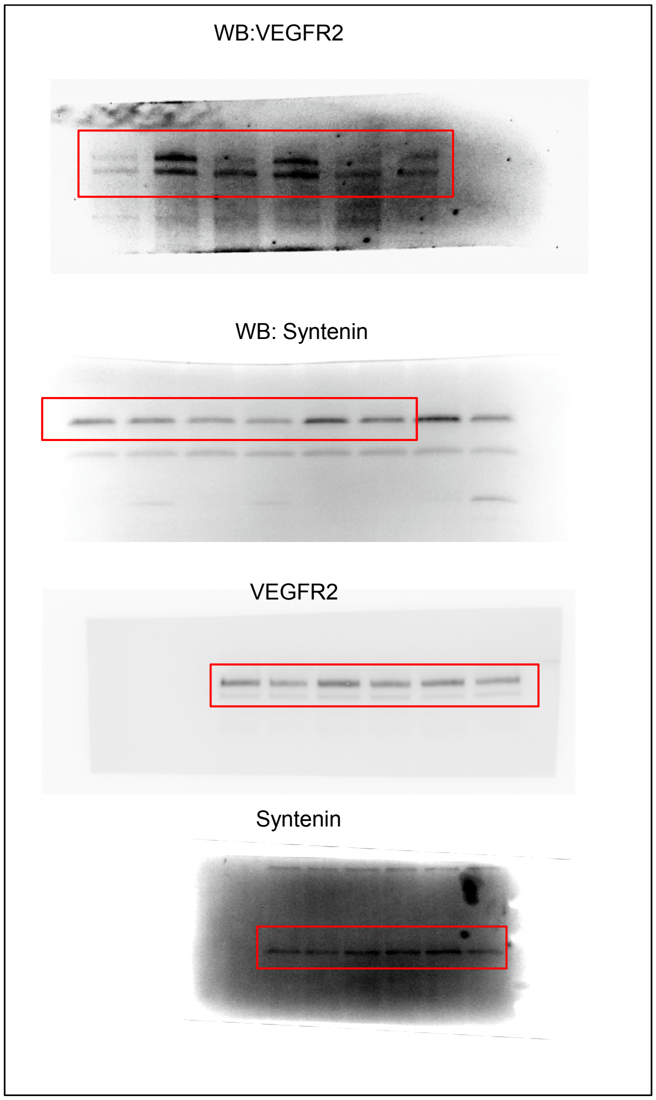

Fig. 5f

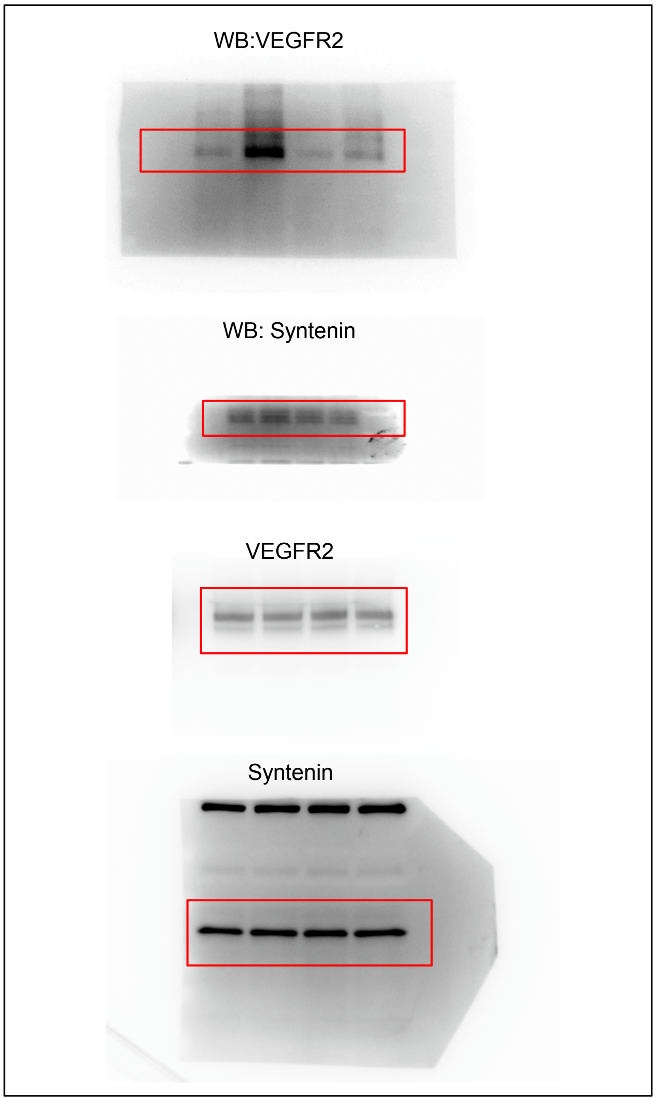

Supplementary Fig. 4h

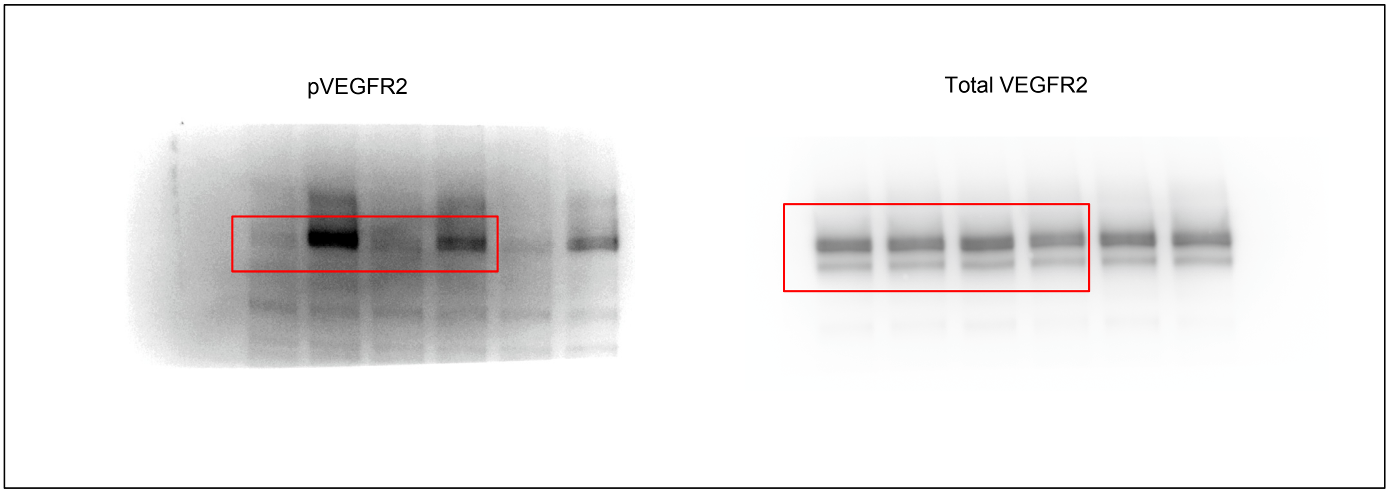

Supplementary Figure 11 | Uncropped western blot images (continued). Membranes for figure 5e,f and supplementary figure 4h

Supplementary Fig. 5a

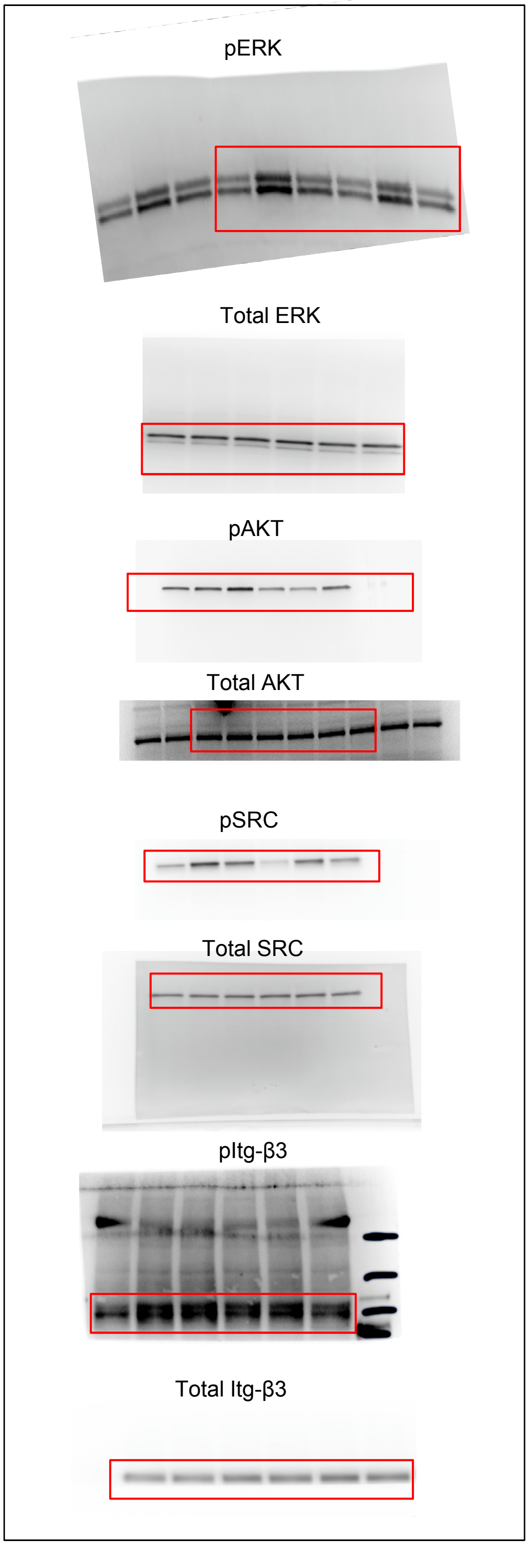

Supplementary Fig. 5f

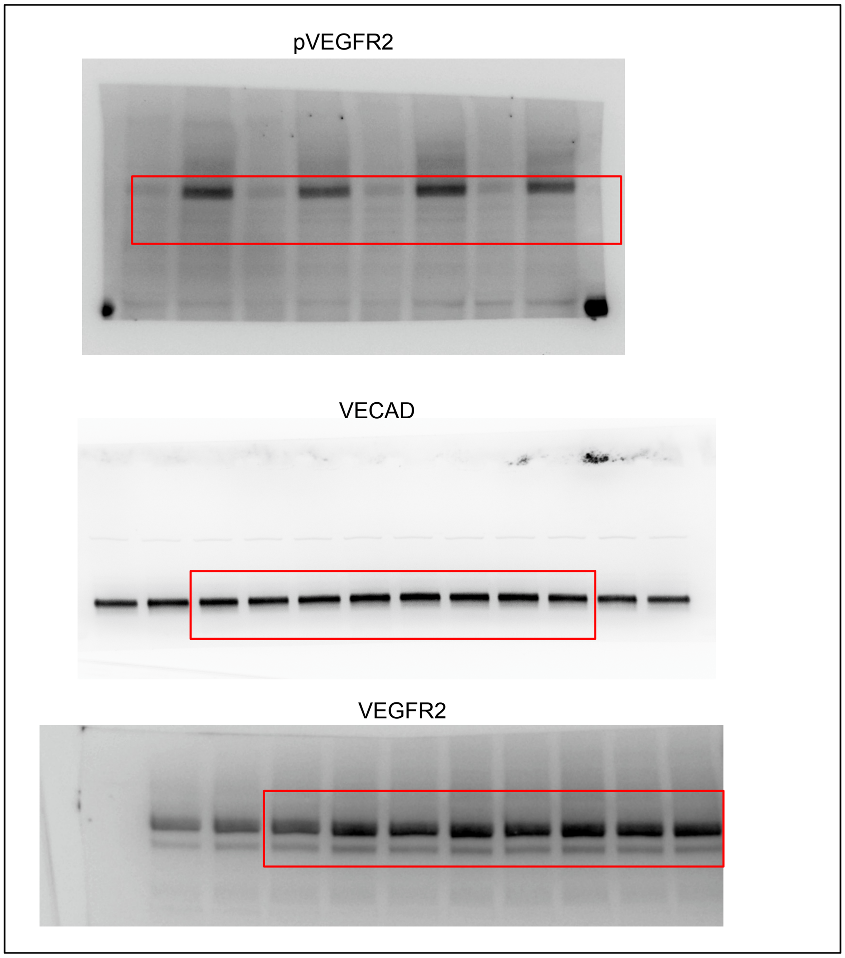

Supplementary Fig. 6c

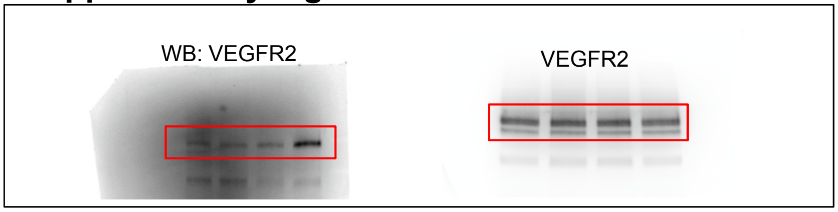

Supplementary Fig. 6d

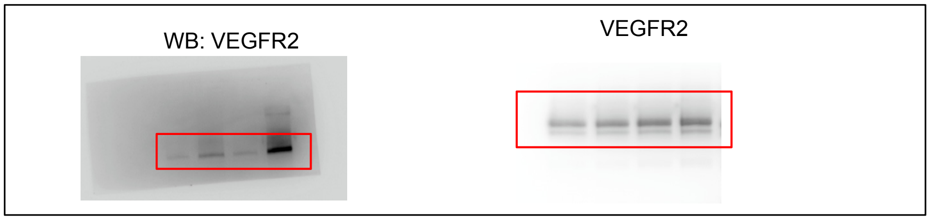

Supplementary Fig. 6f

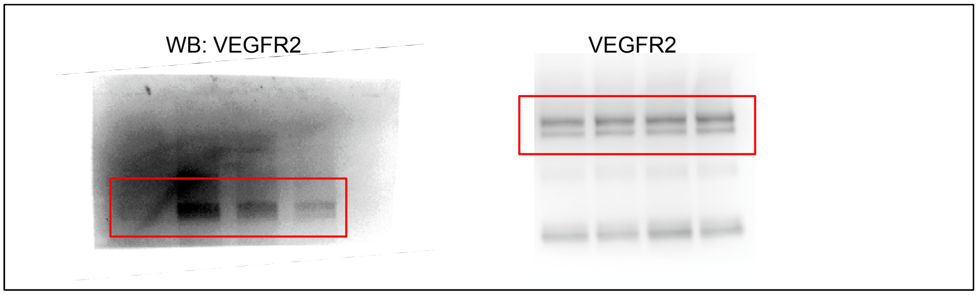

Supplementary Figure 12 | Uncropped western blot images (continued). Membranes for supplementary figures 5a,f and 6c,d,f

Construct Name	Sequence *	Mutations
Ad-Sdc1	Sdc1 (Full length)	
Ad-Sdc2	Sdc2 (Full length)	
Ad-Sdc2$^{no\text{-}tag}$	Mouse Sdc2 (Full length)<br>no tags added	
Ad-Sdc2<br>(C-term-HA)	Human Sdc2 (full length)<br>C-terminal HA-Tag between V-domain and PDZ-binding domain	
Ad-Sdc2<br>(N-term-Flag)	Human Sdc2 (full length)<br>N-terminal Flag-Tag after signal peptide	
Ad-Sdc2$^{\Delta GAG}$	Sdc2 (Full length)	S41A, S55A, S57A
Ad-Sdc3	Sdc3 (Full length)	
Ad-Sdc4	Sdc4 (Full length)	
Ad-Sdc4$^{no\text{-}tag}$	Mouse Sdc4 (Full length)<br>no tags added	
Ad-Sdc4<br>(C-term-HA)	Sdc4 (full length)<br>C-terminal HA-Tag between V-domain and PDZ-binding domain	
Ad-Sdc2^{IN}/Sdc4^{EX}	Sdc2 (ED) + Sdc4 (Tm + ICD)	
Ad-Sdc4^{IN}/Sdc2^{EX}	Sdc4 (ED) + Sdc2 (Tm + ICD)	
Ad-Sdc2^{D1}/Sdc4^{D2}	Sdc2 (D1) + Sdc4 (D2 + Tm + ICD)	
Ad-Sdc4^{D1}/Sdc2^{D2}	Sdc4 (D1) + Sdc4 (D2 + Tm + ICD)	
Ad-Sdc2^{D1}	D2 deletion in Sdc2	
Ad-Sdc4^{D1}	D2 deletion in Sdc4	

Supplementary Table 1 | List of constructs. * If not explicitly indicated, all constructs express the human sequence with an N-terminal HA-tag after signal peptide

Gene	Forward	Reverse
ANGPT2 (mouse)	TCGCTGGTGAAGAGTCCAAC	GTCAAACCACCAGCCTCCTG
EGR3 (mouse)	TGGCTACAGAGAATGTGATGGA	TCCCAAGTAGGTCACGGTCT
GAPDH (human)	GAGTCAACGGATTTGGTCGT	GACAAGCTTCCCGTTCTCAG
GAPDH (mouse)	AACTTTGGCATTGTGGAAGG	ACACATTGGGGGTAGGAACA
NR4A2 (mouse)	GCACTTCGGCGGAGTTGAA	TACTGCGCCTGAACACAAGG
NRP1 (mouse)	GGAGCTACTGGGCTGTGAAG	CCTCCTGTGAGCTGGAAGTC
RCAN1 (mouse)	GCCACCTGGACCCGC	TGTGTAAAGTCTGAGCAAAATACAA
SDC2 (human)	GTGGATCCTGCTCACCTTG	TTATCAGATGTCAGCTCTGCTCTC
SDC2 (mouse)	CTAGTGCTGCTTCCCCCAAA	CAGCAATGACGGCTGCTAGA
VECAD (mouse)	GGACAAGATCAGCTCCTCCA	CGGTTCACGTTGGACTTGAT
VEGFR2 (mouse)	AAGTGTGCGACCCCAAATTC	ACCATCCCACTGTCTGTCTG

Supplementary Table 2 | List of primers in alphabetic order (species indicated in parenthesis)
