# Supplementary material for: N-terminal syndecan-2 domain selectively enhances 6-O heparan sulfate chains sulfation and promotes VEGFA165-dependent neovascularization
Source: Nat Commun. 2019 Apr 5;10:1562. doi: 10.1038/s41467-019-09605-z (PMC6450910; doi:10.1038/s41467-019-09605-z)
Supplement: Supplementary file 3 — Reporting Summary [file 41467_2019_9605_MOESM3_ESM.pdf]

nature research

Corresponding author(s): Michael Simons

Last updated by author(s): Mar 13, 2019

Reporting Summary

Nature Research wishes to improve the reproducibility of the work that we publish. This form provides structure for consistency and transparency in reporting. For further information on Nature Research policies, see Authors & Referees and the Editorial Policy Checklist.

Statistics

For all statistical analyses, confirm that the following items are present in the figure legend, table legend, main text, or Methods section.

n/a	Confirmed	
☐	☒	The exact sample size (*n*) for each experimental group/condition, given as a discrete number and unit of measurement
☐	☒	A statement on whether measurements were taken from distinct samples or whether the same sample was measured repeatedly
☐	☒	The statistical test(s) used AND whether they are one- or two-sided<br>*Only common tests should be described solely by name; describe more complex techniques in the Methods section.*
☒	☐	A description of all covariates tested
☐	☒	A description of any assumptions or corrections, such as tests of normality and adjustment for multiple comparisons
☐	☒	A full description of the statistical parameters including central tendency (e.g. means) or other basic estimates (e.g. regression coefficient) AND variation (e.g. standard deviation) or associated estimates of uncertainty (e.g. confidence intervals)
☐	☒	For null hypothesis testing, the test statistic (e.g. *F*, *t*, *r*) with confidence intervals, effect sizes, degrees of freedom and *P* value noted<br>*Give P values as exact values whenever suitable.*
☒	☐	For Bayesian analysis, information on the choice of priors and Markov chain Monte Carlo settings
☒	☐	For hierarchical and complex designs, identification of the appropriate level for tests and full reporting of outcomes
☒	☐	Estimates of effect sizes (e.g. Cohen's *d*, Pearson's *r*), indicating how they were calculated

Our web collection on statistics for biologists contains articles on many of the points above.

Software and code

Policy information about availability of computer code

Data collection	No softwares were used
Data analysis	ImageJ, GraphPad Prism 7, Excel 2016

For manuscripts utilizing custom algorithms or software that are central to the research but not yet described in published literature, software must be made available to editors/reviewers. We strongly encourage code deposition in a community repository (e.g. GitHub). See the Nature Research guidelines for submitting code & software for further information.

Data

Policy information about availability of data

All manuscripts must include a data availability statement. This statement should provide the following information, where applicable:

- Accession codes, unique identifiers, or web links for publicly available datasets
- A list of figures that have associated raw data
- A description of any restrictions on data availability

All data presented in this manuscript are available from corresponding author upon reasonable request

Field-specific reporting

Please select the one below that is the best fit for your research. If you are not sure, read the appropriate sections before making your selection.

☒ Life sciences ☐ Behavioural & social sciences ☐ Ecological, evolutionary & environmental sciences

For a reference copy of the document with all sections, see nature.com/documents/nr-reporting-summary-flat.pdf

Life sciences study design

All studies must disclose on these points even when the disclosure is negative.

Sample size	No statistical method were used to determine sample size
Data exclusions	No data exclusion
Replication	All details of replications are reported in figure legends
Randomization	Not applicable
Blinding	Analysis of in-vivo vascular phenotypes and disaccharide analysis were confirmed in blinded-manner

Reporting for specific materials, systems and methods

We require information from authors about some types of materials, experimental systems and methods used in many studies. Here, indicate whether each material, system or method listed is relevant to your study. If you are not sure if a list item applies to your research, read the appropriate section before selecting a response.

Materials & experimental systems

n/a	Involved in the study	
☐	☒	Antibodies
☐	☒	Eukaryotic cell lines
☒	☐	Palaeontology
☐	☒	Animals and other organisms
☒	☐	Human research participants
☒	☐	Clinical data

Methods

n/a	Involved in the study	
☒	☐	ChIP-seq
☒	☐	Flow cytometry
☒	☐	MRI-based neuroimaging

Antibodies

Antibodies used	List of antibodies with application and dilutions (IHC, immunohistochemistry; WB; western blot): pAKT T308 (WB 1:1000, Cell Signaling #4056), Total AKT (WB 1:1000, Cell Signaling # 4691), ERG (IHC 1:100, Abcam #ab92513), pERK (WB 1:1000, Cell Signaling #9106,), Total ERK (1:2000, Cell Signaling #9102, WB), FGFR1 (Cell signaling #9740, WB 1:1000), Flag-tag (WB 1:500, Sigma-Aldrich F1804), HA-tag (WB 1:1000, Cell signaling #3724), NRP1 (Cell signaling #3725, WB, 1:1000), pIntegrin (Itg)-β3 Y759 (WB 1:200, Santa Cruz #sc136458), Total Integrin-β3 (WB 1:1000, Cell Signaling #13166) PECAM1 (IHC 1:200, BD Pharmingen #553370), Sdc2-mouse (IHC 1:200, LSBIO #LS-B2981), Sdc2-mouse (WB 1:200, R&D #AF6585 - polyclonal raised against mouse Sdc2 ED), Sdc2-human (WB 1:200, R&D # AF2965 , polyclonal raised against mouse human ED), Sdc4 (WB 1:500, Abcam #ab24511 – monoclonal against C-terminal domain), pSRC Y416 (WB 1:1000, Cell Signaling #6943), Total Src (WB 1:1000, Cell Signaling #2109), pVEGFR2 Y1175 (WB 1:1000, Cell Signaling #2478), Total VEGFR2 (WB 1:1000, Cell Signaling #2479), VE-Cad (WB 1:200, Santa Cruz #sc-6458 C-19 clone), GFP (Santa Cruz #sc-9996, WB, 1:200), Syntenin (WB 1:500, Abcam #ab19903).
Validation	Sdc2 antibodies were validated in this study. All other antibodies were extensively validated in previously published papers and by suppliers.

Eukaryotic cell lines

Policy information about cell lines

Cell line source(s)	Human Umbilical Vein Endothelial Cells (HUVEC) were obtained from Yale VBT tissue-culture core laboratory.<br>Mouse primary endothelial cells were isolated from freshly collected mouse heart or lung tissues<br>MS-1 cells were purchased from ATCC (CRL-2229).<br>293A cell were purchased from Invitrogen (R70507)
Authentication	Endothelial cells are fully authenticated with specific markers in this paper. Other cell types are characterized by supplier and functionally authenticated in this paper.
Mycoplasma contamination	None
Commonly misidentified lines (See ICLAC register)	None

Animals and other organisms

Policy information about studies involving animals; ARRIVE guidelines recommended for reporting animal research

Laboratory animals	Species: Mouse (Mus musculus)<br>Strains: Sdc2 Knockout-first transgenic mice (Sdc2tm1a(KOMP)Wtsi). Sdc2 (sdc2-/-) and Sdc4 (sdc4-/-) global knockout mice. Sdc2 conditional transgenic mice (sdc2fl/fl). Sdc2 endothelial-specific knockout mice (sdc2icdh5 or Sdc2ipdgfb). All animals were back-crossed at least 10 generation to a C57Black6/J strain<br>Sex: males<br>Age: neonates and adults
Wild animals	*Provide details on animals observed in or captured in the field; report species, sex and age where possible. Describe how animals were caught and transported and what happened to captive animals after the study (if killed, explain why and describe method; if released, say where and when) OR state that the study did not involve wild animals.*
Field-collected samples	*For laboratory work with field-collected samples, describe all relevant parameters such as housing, maintenance, temperature, photoperiod and end-of-experiment protocol OR state that the study did not involve samples collected from the field.*
Ethics oversight	All mouse experimental protocols have been approved by the Institutional Animal Care & Use Committee (IACUC) at Yale University. The authors have complied with all relevant animal testing and research ethical regulations.

Note that full information on the approval of the study protocol must also be provided in the manuscript.
